# Compulsivity Across the Pathological Misuse of Drug and Non-Drug Rewards

**DOI:** 10.3389/fnbeh.2016.00154

**Published:** 2016-08-03

**Authors:** Paula Banca, Neil A. Harrison, Valerie Voon

**Affiliations:** ^1^Behavioral and Clinical Neuroscience Institute, University of CambridgeCambridge, UK; ^2^Brighton and Sussex Medical School, University of SussexBrighton, UK; ^3^Department of Psychiatry, University of CambridgeCambridge, UK; ^4^Cambridgeshire and Peterborough NHS Foundation TrustCambridge, UK

**Keywords:** addiction, alcohol dependence, binge eating disorder, compulsivity, reversal learning, set-shifting

## Abstract

Behavioral adaptation is required for the successful navigation of a constantly changing environment. Impairments in behavioral flexibility are commonly observed in psychiatric disorders including those of addiction. This study investigates two distinct facets of compulsivity, namely reversal learning and attentional set shifting, implicating orbitofrontal and lateral prefrontal regions respectively, across disorders of primary and secondary rewards. Obese subjects with and without binge eating disorder (BED), individuals with compulsive sexual behaviors (CSB), alcohol dependence (AD) and pathological video-gaming (VG) were tested with two computerized tasks: the probabilistic reversal task (trials to criterion and win-stay/lose-shift errors) and the intra/extra-dimensional set shift task (IED). Individuals with AD and pathological VG were slower at reversal learning irrespective of valence, with AD subjects more likely to perseverate after losses. Compared to obese subjects without BED, BED subjects were worse at reversal learning to wins but better at losses highlighting valence effects as a function of binge eating. CSB subjects demonstrated enhanced sensitivity to reward outcomes with faster acquisition and greater perseveration with higher magnitude rewards. We further show an impairment in attentional set shifting in individuals with BED and AD relative to healthy volunteers (HV). This study provides evidence for commonalities and differences in two distinct dimensions of behavioral inflexibility across disorders of compulsivity. We summarize studies on compulsivity subtypes within this same patient population. We emphasize commonalities in AD and BED with impairments across a range of compulsivity indices, perhaps supporting pathological binge eating as a form of behavioral addiction. We further emphasize commonalities in reversal learning across disorders and the crucial role of valence effects. These findings highlight the role of behavioral inflexibility and compulsivity as a relevant domain in defining dimensional psychiatry and the identification of relevant cognitive endophenotypes as targets for therapeutic modulation.

## Introduction

The ability to adjust behaviors is crucial for optimal navigation in a constantly changing environment. Behavioral inflexibility has wide individual variability and is a typical cognitive feature in disorders of addiction, commonly linked to compulsivity. Compulsivity has been suggested to be a heterogeneous construct, which can be divided into subtypes with distinct but overlapping neural networks (Dalley et al., 2011). Here we focus on two specific facets of compulsivity namely reversal learning and attentional set shifting. Reversal learning measures the capacity to flexibly switch choices with changes in contingencies and implicates orbitofrontal cortices whereas attentional set shifting is the ability to shift response sets to a previously irrelevant dimension and implicates lateral prefrontal cortices (Kringelbach and Rolls, 2003; McAlonan and Brown, 2003; O’Doherty et al., 2003; Hornak et al., 2004; Robbins, 2007). Deficits in these markers of cognitive inflexibility have been classically studied across species (for review on reversal learning see Izquierdo et al., 2016; and on attentional set shifting see Brown and Tait, 2016; Izquierdo et al., 2016). Many of these studies have assessed these two markers in disorders of addiction to drug rewards but, although there is a large number of studies on cognitive flexibility in experimental species for natural rewards, fewer studies in humans have focused on disorders of natural rewards. We investigate these cognitive processes and the influence of the role of outcome valence across disorders characterized by the pathological misuse of drug and non-drug rewards. In keeping with the trend towards dimensional psychiatry (Insel et al., 2010), we seek to compare the cross-diagnostic neurocognitive profile focusing on compulsivity to enhance our understanding of current psychiatric disorder classifications (Robbins et al., 2012).

In reversal learning paradigms, subjects are required to adapt their internal representations and choices when feedback demonstrates an outcome change (Cools et al., 2002; Clarke et al., 2005). Reversal learning is strongly impaired in cocaine use disorders (Fillmore and Rush, 2006; Ersche et al., 2008; Camchong et al., 2011; Fernández-Serrano et al., 2012). Data from amphetamine is less consistent. One study has found unimpaired reversal learning in amphetamine and opiate users (Ersche et al., 2008) but others have shown that even brief exposure to methamphetamine results in selective impairments on reversal tasks in rats (Cheng et al., 2007; White et al., 2009; Izquierdo et al., 2010; for review of reversal learning in addiction see Izquierdo and Jentsch, 2012). Reversal learning impairments are also not prominent in alcohol dependence (AD). Impaired discrimination reversal has been shown following aversive eye-blink conditioning in abstinent chronic alcoholics (Fortier et al., 2008) and AD individuals have also been shown to be slower at reversal learning in a deterministic reversal task although there were no differences in the primary outcome measures (Vanes et al., 2014). Impairments have also been found in pathological gambling using a probabilistic reversal learning task for gain and loss (Patterson et al., 2006; de Ruiter et al., 2009). In obese women, deficits in both acquisition and reversal learning have been shown specific to food cues using an appetitive reversal learning task (Zhang et al., 2014).

The distinct yet related measure of cognitive inflexibility, cognitive set-shifting, as measured by the wisconsin card sorting task (WCST) or Intra-/Extra-dimensional set shifting (IED) task, probes the capacity to switch responding to previously irrelevant stimuli, requiring attentional flexibility. Studies with current amphetamine or methamphetamine users report reduced set shifting in users (Ornstein et al., 2000; Clark et al., 2006) with improvement in performance with prolonged drug abstinence (Toomey et al., 2003; Johanson et al., 2006; van den Hout et al., 2009). In AD individuals, alcohol misuse severity and impairments in set-shifting have previously been shown to be linked to relapse (Pothiyil and Alex, 2013). Impairments in the WCST have been shown particularly in chronic alcoholics with a history in alcoholism of greater than 10 years (Tarter, 1973). Furthermore, set-shifting impairments associated with AD have been shown to not improve after a period of abstinence (Nowakowska et al., 2007).

Poor set-shifting and increased perseveration have also been shown in obese individuals with and without binge eating disorder (BED; Duchesne et al., 2010; Wu et al., 2014) and in children and adolescent with excess weight (Cserjési et al., 2007; Verdejo-García et al., 2010) using the WCST and the Trail-making test. Contradictory results have been shown in pathological gambling, where both reduced set-shifting in the IED task (Grant et al., 2011) and normal performance on the WCST task (Goudriaan et al., 2006) has been demonstrated.

Here we investigate the behavioral markers of compulsivity focusing on reversal learning and set shifting across several different addictive disorders. The reversal learning task was modified to investigate whether different pathologies would show different sensitivity to reward and loss magnitude outcomes. This is important considering that several studies have provided evidence for dissociable neural responses to reward and punishment outcomes (O’Doherty et al., 2001; Remijnse et al., 2005; Robinson et al., 2010). We compare abstinent AD individuals and obese subjects with and without BED with their own healthy volunteers (HV). The rodent model of sucrose binge eating (Avena et al., 2008), which has been showing many similarities with models of substance-use disorders, has been suggesting the binge-eating pattern of food intake to be a crucial subtype that differentiates obese individuals. We subdivided our obese group into individuals with and without binge eating behaviors to assess the relationship between this important feature (pattern of food intake) and the constructs assessed by these cognitive tasks. On an exploratory basis, we also compare individuals with pathological video-gaming (VG) behaviors and with compulsive sexual behaviors (CSB) with their own matched healthy controls. We hypothesize that AD and obese individuals with, but not without, BED will be characterized by impairments in reversal learning and set shifting cognitive processes. Furthermore, we expect to see a differential influence of valence (reward/loss) in behavior across disorders, similarly to our previous findings in risk-taking reporting that pathological choices are influenced by factors of valence, probability, and magnitude (Voon et al., 2015c).

## Materials and Methods

### Recruitment

Healthy controls and AD, Obese with and without BED, VG and CSB individuals were recruited via community-and university-based advertisements in Cambridge. All subject groups were age- and gender-matched with their own HV. All volunteers completed the same basic interview assessment to detect all types of compulsive behaviors and exclude other psychiatric disorders. All diagnoses were made by a psychiatrist. Obese individuals had a body mass index (BMI) of ≥30 and those with BED fulfilled DSM-IV-TR BED criteria in addition (American Psychiatric Association, 2000). Individuals with AD fulfilled DSM-IV criteria for AD, and were abstinent for at least 2 weeks to 1 year prior to testing. VG participants were included if they fulfilled VG criteria adapted from DSM-IV pathological gambling criteria (American Psychiatric Association, 2000) in keeping with other published studies (Gentile et al., 2011). CSB individuals were also recruited via advertisements placed on internet sites including on Reddit and from therapist referrals. CSB subjects were screened using the internet sex screening test (ISST; Delmonico and Miller, 2003) and an exhaustive experimenter-designed questionnaire which included items pertaining to age of onset, frequency, duration, attempts to control use, abstinence, patterns of use, treatment and negative consequences. CSB participants were interviewed by a psychiatrist to confirm they fulfilled two sets of diagnostic criteria for CSB (proposed diagnostic criteria for Hypersexual Disorder; criteria for sexual addiction; Carnes et al., 2001; Kafka, 2010; Reid et al., 2012), focusing on compulsive use of online sexually explicit material. These criteria emphasize failure to cut down or control sexual behaviors, including consumption of pornography, despite social, financial, psychological and academic or vocational problems. Detailed description of CSB symptoms are described in Voon et al. (2014).

All the participants were included if they were aged ≥18 years and excluded if they had a current major depression or a history of other severe psychiatric disorder (e.g., bipolar affective disorder or schizophrenia) or a current substance use disorder including regular cannabis use. Participants were also excluded if they tested positive for a drug urine screen (including cannabis) or alcohol breathalyzer test on the day of testing. CSB and their matched HV were male and heterosexual.

### Procedure

Following provision of written consent, all participants underwent urine and breathalyzer tests on the day of testing. All participants completed the Beck Depression Inventory-II (BDI-II; Beck et al., 1996) to assess depressive symptoms, the UPPS-P Impulsive Behavior Scale to assess impulsivity (Whiteside and Lynam, 2001), the State Trait Anxiety Inventory (STAI; Spielberger et al., 1983) to assess depression and anxiety, and the ADs Identification Test (ADIT; Saunders et al., 1993). The National Adult Reading Test (NART; Nelson, 1982) provided an index of premorbid IQ. Subjects were screened for comorbid psychiatric disorders with the Mini International Neuropsychiatric Inventory (MINI; Sheehan et al., 1998). All participants refrained from consuming alcohol for at least 24 h prior to their study visit. The study was approved by the University of Cambridge Research Ethics Committee. Subjects were remunerated at a rate of £7.50 per hour including travel costs, with an additional £5 contingent on task performance.

### Behavioral Measures

#### Probabilistic Reversal Task

**Figure 1:**
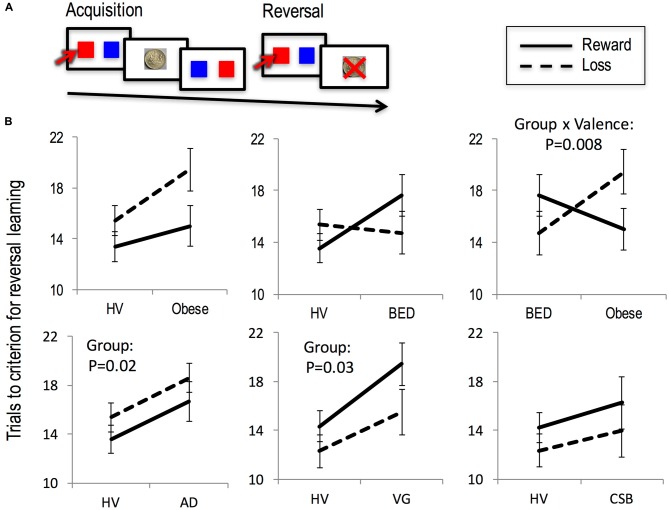
**Reversal learning. (A)** Probabilistic reversal learning task. **(B)** The number of trials to criterion for reversal learning in the context of reward (solid line) and loss (dashed line) is depicted for Obese subjects with and without binge eating disorder (BED), alcohol dependence (AD), compulsive sexual behavior (CSB) and pathological video-gaming (VG), each with their own matched healthy volunteer (HV) group.

The probabilistic reversal task comprised two phases, acquisition and reversal, with three conditions varying by magnitude of reward or loss outcome (reward, neutral and loss) in each phase. In the acquisition phase, subjects chose from one of the three stimulus-pairs associated with the following probabilistic outcomes: Loss (Stimulus A: *P* = 0.30, Win +£1/*P* = 0.70, Lose −£2 (mean −£1.1); Stimulus B: *P* = 0.70, Win +£1/*P* = 0.30, Lose −£2 (mean +£0.1)), Neutral (Stimulus C: *P* = 0.70, Win +£1/*P* = 0.30, Lose −£1 (mean +£0.4); Stimulus D: *P* = 0.30, Win +£1/*P* = 0.70, Lose −£1 (mean −£0.4)) or Reward (Stimulus E: *P* = 0.70, Win +£2/*P* = 0.30, Lose −£1 (mean +£1,1); Stimulus F: *P* = 0.30, Win +£2/*P* = 0.70, Lose −£1 (mean −£0.1); Figure 1A). Subjects were shown and chosen from one stimuli-pair at a time followed by the outcome. After 30 trials per condition in the acquisition phase, the contingencies for each stimulus-pair switched and were followed by 30 trials per condition in the reversal phase (e.g., Stimulus A: *P* = 0.70, Win +£1/*P* = 0.3, Lose −£2; Stimulus B: *P* = 0.30, Win +£1/*P* = 0.70, Lose −£2). There were a total of 180 trials (60 trials per condition). The position of the stimuli within each stimulus-pair was counterbalanced on either side of the screen. The stimuli-pairs of the different conditions were randomly presented; thus, subjects were exposed to different trial sequences.

Subjects were given 10 practice trials of a stimuli-pair in which one stimulus was associated with *P* = 0.70, Win +£1/*P* = 0.30, Lose −£1 and the other stimulus associated with *P* = 0.30, Win +£1/*P* = 0.70, Lose −£1). The stimulus phase was shown for 2.5 s during which the participants needed to respond. Subjects indicated a response with their dominant hand pressing the left arrow on the keyboard for the stimulus on the left and the right arrow on the keyboard for the stimulus on the right. The stimulus phase was followed by a 1 s outcome phase with the words “You WON!!” and an image of a £2 or £1 coin or “You LOST!!” and an image of a large red “X” over the £2 or £1 coin. If subjects were too slow, this was followed by the words: “You were too slow. Respond faster”. The trial was followed by a variable inter-trial interval of a mean of 0.75 s varying between 0.5 and 1 s.

Participants were told they would choose from three different pairs of symbols and that one symbol within each pair was more likely to be associated with winning money or not losing money. They were also told that at some point the relationship between the symbols and the likelihood of winning and not losing money might change. Participants were instructed that their goal was to make as much money as possible of which a proportion would be paid to them at the end of the study.

The primary outcome measure of an index of a learning rate was the number of trials to criterion of four correct sequential choices within each condition (Voon et al., 2015a; Morris et al., 2016). This outcome provides an index of how quickly subjects learn contingencies during acquisition and reversal. As a secondary analysis, the outcome measure of win-stay (the proportion of trials in which the same stimulus was selected following a win outcome = win-stay/(win-stay + win-shift)) or lose-shift (the proportion of trials in which the opposite stimulus was selected following a loss outcome), were assessed. This outcome provides an index of sensitivity to outcome and behavioral strategy as a function of the most recent outcome. Outcome measures were calculated separately for acquisition and reversal phases. The task was coded in E-prime Version 2 and took approximately 13 min to get completed.

#### Intra/Extra-Dimensional Set Shift Task (IED)

**Figure 2:**
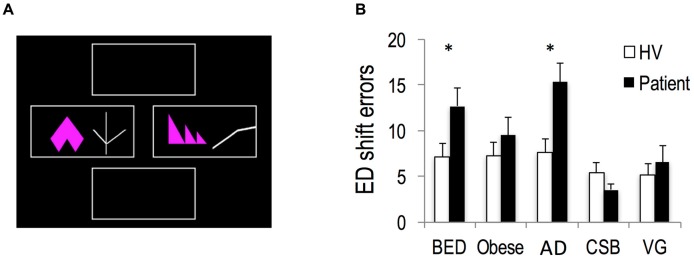
**Attentional set-shifting. (A)** Intra- and extra-dimensional set shifting task. **(B)** Extra-dimensional (ED) shift errors in Obese subjects with and without BED, subjects with AD, CSB and pathological VG compared to each groups’ own matched healthy volunteers (HV). *p < 0.05.

The IED is a deterministic reward reversal task from the Cambridge Neuropsychological Test Automated Battery (CANTAB; Sahakian and Owen, 1992; Figure 2). It is performed on a touch screen monitor. The task is composed of nine stages beginning with a simple discrimination and its reversal of one dimension (pink shapes). Compound discrimination and its reversal are then tested with the addition of another dimension (adjacent or overlying white lines). Success is dependent on responding to the previous relevant dimension and on ignoring the new, irrelevant dimension. On any given trial participants choose which of a pair of stimuli are “correct” by touching that stimulus. In stage 1, the stimuli varied along only one dimension (pink shapes) after six correct choices and the next stage (stage 2) commences in which contingencies reverse such that the other paired stimulus is correct. Stage 3 introduces a new dimension (adjacent white lines) to get the subject familiar with a compound stimulus. To succeed, participants had to continue responding to the correct stimulus of previous stages. Stage 4 and the subsequent stages were also compound but the two stimulus from the different dimensions were superimposed (the white lines overlying the pink shapes). The contingencies again remained unchanged from those for the previous stage. A reversal then occurred in stage 5. Stage 6 introduced new stimuli for both dimensions, with the relevant dimension unchanged. It required the subject to continue to attend to the shape dimension and learn which of the two new exemplars is correct (“intra-dimensional shift”). Following reversal (stage 7), a final set of compound stimuli are presented. In stage 8, the subject is now required to attend to the previously-redundant dimension of line (“extra-dimensional shift”). Finally, contingencies were reversed to the previously incorrect exemplar of the new dimension (stage 9). If a subject fails to complete six correct responses in a row, in any given stage, the test terminates. Of the nine blocks, four (2, 5, 7 and 9) represent reversal stages. The task duration is approximately 7 min. The primary outcome measure was the number of errors in the extra-dimensional (ED) shift stage. This outcome provides an index of set shifting or how many errors occurred prior to learning the shift to the previously redundant dimension.

### Statistics

The data was inspected for outliers and normality of distribution tested using Shapiro-Wilkes test. Subject characteristics were analyzed using independent *t*-tests. As the data for reversal trials to criterion was left-skewed, the data was transformed by squaring. As the data for acquisition trials to criterion was right-skewed, the data was transformed by square root. The number of trials to criterion of the reversal phase was analyzed using a mixed measures ANOVA with Group as a between-subjects factor and Valence (Reward and Loss) as a within-subjects factor. The number of trials to criterion for the acquisition phase was similarly assessed. For the reversal learning task, the outcome of Win-Stay and Lose-Shift were separately assessed using a mixed measures ANOVA with Group as a between-subjects factor, Valence condition (Reward, Loss and Neutral) and Phase (Acquisition vs. Reversal) as a within-subjects factor. For the IED, the primary outcome of errors during the ED shift was assessed between groups using independent *t*-tests. A value of *p* < 0.05 was taken to indicate statistical significance.

## Results

Age- and gender-matched HVs were compared with each group (AD: *N* = 32; Obese: *N* = 31; BED: *N* = 32; VG: *N* = 26; CSB: *N* = 25). Subject characteristics, behavioral measures and sample sizes are reported in Tables 1, 2. AD subjects had the following alcohol use (weeks abstinent 16.53 (SD 16.99); years of dependence 12.92 (SD 8.17); Units/day 28.51 (SD 14.32). Three subjects in the AD group were on the following medications (acamprosate 2; disulfiram 1).

**Table 1:** **Subject characteristics and behavioral measures**.

	AD	HV-AD	*t*	Obese BED	HV-BED	*t*	Obese	HV-Obese	*t*
			*p*			*p*			*p*
*N*	32	64		32	64		31	62
Age	41.37	42.65	0.503	42.81	43.49	0.308	43.89	43.15	0.338
	(11.44)	(11.91)	0.616	(8.63)	(10.88)	0.759	(9.63)	(10.13)	0.737
Age range	21–66	20–62		26–54	23–55		27–66	23–63	
Males (N)	19	38		14	28		19	38	
IQ	114.11	115.63	1.126	115.38	114.72	0.439	115.79	114.88	0.676
	(6.81)	(5.93)	0.263	(6.81)	(7.01)	0.662	(6.51)	(6.73)	0.501
BDI	12.73	5.95	4.343	12.11	5.98	4.686	7.01	5.98	0.831
	(9.18)	(6.01)	<0.001	(6.49)	(5.81)	<0.001	(6.03)	(5.39)	0.406
UPPS	153.95	122.14	6.061	131.95	122.83	1.926	128.39	122.85	1.149
	(20.91)	(25.72)	<0.001	(19.85)	(22.79)	0.057	(19.72)	(22.92)	0.254
BMI				34.72	22.87	13.628	32.71	23.16	14.944
				(5.63)	(2.91)	<0.001	(3.59)	(2.73)	<0.001
BES				24.95	6.31	13.222	8.81	6.99	1.195
				(7.12)	(6.19)	<0.001	(7.31)	(6.89)	0.235

*Abbreviations: AD, Alcohol dependent subjects; BED, Binge eating disorder; HV, healthy volunteers; N, number of subjects; BDI, Beck Depression Inventory; UPPS, UPPS Impulsive Behavior Scale; BMI, Body Mass Index; BES, Binge Eating Severity*.

**Table 2:** **Subject characteristics and behavioral measures in pathological videogaming and compulsive sexual behaviors**.

	VG	HV -VG	*t*	CSB	HV -CSB	*t*
			*p*			*p*
*N*	26	52		25	50
Age	24.69	22.91	1.441	28.5	27.88	0.300
	(5.90)	(4.73)	0.154	(8.57)	(7.52)	0.764
Age range	18–48	18–45		19–53	18–51	
Males (N)	13	26		25	50	
IQ	119.80	117.58	1.843	111.93	113.15	0.666
	(4.33)	(5.32)	0.069	(6.71)	(7.83)	0.508
BDI	7.61	4.35	2.600	7.18	5.31	1.347
	(5.28)	(5.19)	0.011	(6.03)	(5.48)	0.182
UPPS	136.60	128.49	1.583	151.72	129.27	4.256
	(19.98)	(21.95)	0.117	(18.33)	(22.94)	<0.001

*Abbreviations: VG, pathological videogaming; CSB, compulsive sexual behavior; HV, healthy volunteers; N, number of subjects; BDI, Beck Depression Inventory; UPPS, UPPS Impulsive Behavior Scale*.

### Probabilistic Reversal Task

#### Obese Subjects With and Without BED

##### Trials to criterion

In the Reversal phase (Figure 1) in BED (*N* = 32) subjects compared to HVs (*N* = 64) there was no main effect of Valence (*F*_(1,94)_ = 0.12, *p* = 0.726), or Group (*F*_(1,94)_ = 1.19, *p* = 0.278) or Group × Valence interaction (*F*_(1,94)_ = 3.04, *p* = 0.085).

In the Reversal phase, Obese subjects (*N* = 31) compared to HV (*N* = 62) presented a main effect of Valence (*F*_(1,91)_ = 7.31, *p* = 0.008), a trend towards a Group effect (*F*_(1,91)_ = 3.53, *p* = 0.063) and no interaction effect (*F*_(1,91)_ = 1.77, *p* = 0.186).

In the comparison of Obese subjects with and without BED, there was a Group × Valence interaction (*F*_(1,61)_ = 7.60, *p* = 0.008) in which Obese subjects with BED required more trials to reach criterion in the reversal phase to Reward and less to Loss with the opposite observed in Obese subjects without BED. There was no main effect of Group (*F*_(1,61)_ = 0.43, *p* = 0.510) or Valence (*F*_(1,61)_ = 0.79, *p* = 0.377). The interaction effect remained significant with the inclusion of BDI as a covariate of no interest (*p* = 0.023).

In the Acquisition phase, Obese subjects compared to HV (Reward: HV 9.84 (SD 7.84); Obese 10.44 (SD 9.00); Loss: HV 7.93 (SD 6.59); Obese 7.96 (SD 9.00)) presented a main effect of Valence (*F*_(1,91)_ = 2.68, *p* = 0.008) but not effects of Group (*F*_(1,91)_ = 0.12, *p* = 0.726) or interaction effect (*F*_(1,91)_ = 1.07, *p* = 0.745). In the Acquisition phase in BED subjects compared to HV (Reward: HV 9.49 (SD 7.46); BED 9.97 (SD 8.29); Loss: HV 7.73 (SD 6.33); BED 7.36 (SD 6.64)) there were no main effects of Valence (*F*_(1,94)_ = 2.82, *p* = 0.105), Group (*F*_(1,94)_ = 0.285, *p* = 0.595) or Interaction (*F*_(1,94)_ = 0.10, *p* = 0.749).

##### Lose-shift/win-stay

**Figure 3:**
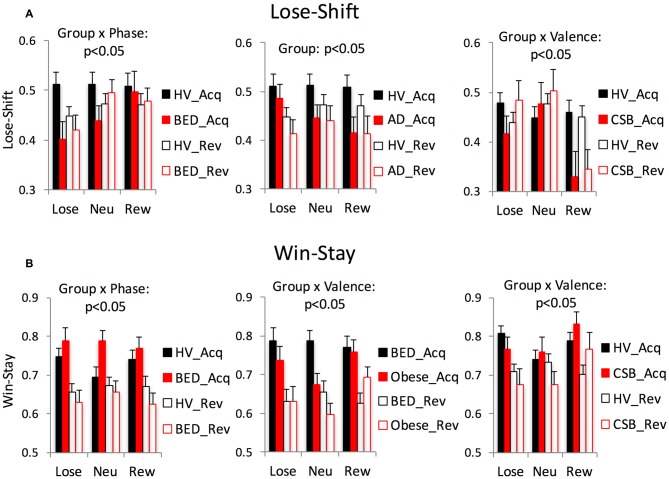
**Lose-shift vs. win-stay.** The significant mixed measures ANOVA findings are shown for Lose-shift **(A)** vs. Win-stay **(B)**. Abbreviations: Neu, neutral; Rew, reward; HV, healthy volunteers; Acq, acquisition; Rev, Reversal; BED, Obese with binge eating disorder; AD, abstinent alcohol dependent subjects; Obese, Obese without BED; CSB, compulsive sexual behaviors.

In the comparison (Table 3; Figure 3) of BED and HV, in both the Lose-Shift and Win-Stay analyses there was an interaction between Group × Phase. In the posthoc analysis, during the Reversal compared to the Acquisition phase, BED subjects had greater Lose-Shift or were more likely to shift or select the opposite stimulus after losing compared to HV. Similarly, during the Reversal compared to Acquisition phase, BED subjects also had lower Win-stay or were less likely to perseverate or stay with the same stimulus after a win compared to HV.

**Table 3:** **Lose-shift and win-stay analysis**.

		**Lose-shift**						**Win-stay**					
		AD	BED	Obese	BED	VG	CSB	AD	BED	Obese	BED	VG	CSB
		vs.	vs.	vs.	vs.	vs.	vs.	vs.	vs.	vs.	vs.	vs.	vs.
		HV	HV	HV	Obese	HV	HV	HV	HV	HV	Obese	HV	HV
Group × Phase	*F*	0.426	**5.009**	2.015	0.688	0.038	1.721	0.113	**8.968**	0.928	3.606	3.361	0.162
	*p*	0.516	**0.028**	0.160	0.410	0.846	0.193	0.737	**0.004**	0.338	0.063	0.070	0.688
Group × Valence	*F*	0.714	1.571	2.789	2.022	0.075	**6.667**	1.062	1.152	2.331	**5.481**	0.799	**3.369**
	*p*	0.493	0.214	0.068	0.142	0.928	**0.002**	0.351	0.322	0.103	**0.007**	0.453	**0.039**
Group × Phase × Valence	*F*	0.412	0.849	0.155	1.121	1.618	1.456	0.220	0.375	0.704	0.112	0.497	1.608
	*p*	0.663	0.432	0.857	0.333	0.204	0.239	0.803	0.689	0.497	0.894	0.610	0.207
Group	*F*	**6.833**	2.356	2.151	0.015	0.365	2.382	3.227	0.217	0.362	0.933	0.457	<0.001
	*p*	**0.011**	0.129	0.146	0.904	0.547	0.243	0.076	0.643	0.549	0.338	0.501	0.987

*Mixed measures ANOVA statistics for Lose-shift and Win-stay are reported for abstinent alcohol dependent (AD) vs. healthy volunteers (HV); Obese subjects with binge eating disorder (BED) vs. HV; Obese subjects without BED (Obese) vs. HV; BED vs. Obese; pathological video-gaming (VG) vs. HV; compulsive sexual behavior (CSB) vs. HV. Significant findings are highlighted in bold*.

In the comparison of obese and HV, there were no significant differences in Lose-Shift or Win-Stay.

In the comparison of BED and Obese, there were no interactions with Group or main Group effect with Lose-Shift. In the Win-Stay analysis comparing BED and Obese, there was a Group × Valence effect, which on posthoc analysis the BED subjects had greater Win-Stay across both Reversal and Acquisition phases or were overall more likely to perseverate after a win in Loss (*p* = 0.024) and Reward (*p* = 0.001) conditions relative to Neutral.

##### Summary

BED subjects compared to obese non-BED subjects showed poorer reversal learning (greater trials to criterion) specifically to reward and not to the loss condition. This converges with a greater Win-Stay in BED or enhanced tendency to perseverate or stay after a win. BED subjects compared to HV showed a greater tendency to switch after any outcome in the reversal phase.

#### Alcohol Dependence

##### Trials to criterion

In the Reversal phase in the AD subjects (*N* = 32) vs. HV (*N* = 64) there was a main effect of Group *F*_(1,94)_ = 5.78, *p* = 0.018) in which AD subjects required more trials to reach criterion compared to HV (Figure 1). These Group results remained significant with a subgroup analysis without the three medicated subjects. There was no effect of Valence (*F*_(1,94)_ = 2.41, *p* = 0.124) or interaction (*F*_(1,94)_ = 0.08, *p* = 0.779). In the Acquisition phase in the AD subjects compared to HV (Reward: HV 9.43 (SD 7.64); AD 9.63 (SD 7.58); Loss: HV 7.51 (SD 6.63); AD 11.61 (SD 9.12)) there was no main effects of Valence (*F*_(1,94)_ = 0.002, *p* = 0.967), Group (*F*_(1,94)_ = 1.70, *p* = 0.196) or interaction (*F*_(1,94)_ = 2.25, *p* = 0.137).

##### Win-stay/lose-shift

In the Lose-Shift analysis, there was a main Group effect (Table 3; Figure 2) in which AD had lower Lose-Shift scores or more likelihood to stay or perseverate after a loss across all trial types compared to HV. The Group effect remained significant with a subgroup analysis without the 3 medicated subjects. There were no differences in the Win-Stay analysis.

##### Summary

AD subjects had slower reversal learning compared to HV and had lower Lose-Shift or a greater tendency to stay or perseverate after a loss.

#### Pathological Videogaming

##### Trials to criterion

In the Reversal phase in the VG subjects (*N* = 26) compared to HV (*N* = 52) there was a main effect of Group (*F*_(1,76)_ = 5.17, *p* = 0.026) in which VG subjects required more trials to reach criterion (Figure 1). There was no Valence (*F*_(1,76)_ = 3.05, *p* = 0.085) or interaction effect (*F*_(1,76)_ = 0.384, *p* = 0.537). In the Acquisition phase (Reward: HV 9.49 (SD 7.51); VG 10.46 (SD 6.36); Loss: HV 7.23 (SD 6.73); VG 7.69 (SD 6.90)) there was an effect of Valence (*F*_(1,76)_ = 7.910, *p* = 0.006) but no effect of Group (*F*_(1,76)_ = 0.86, *p* = 0.356) or interaction (*F*_(1,76)_ = 0.51, *p* = 0.478).

##### Win-stay/lose-shift

There were no Lose-Shift or Win-Stay differences in VG compared to HV.

##### Summary

VG subjects had slower reversal learning compared to HV.

#### Compulsive Sexual Behavior

##### Trails to criterion

In the Reversal phase in the CSB subjects (*N* = 25) compared to HV (*N* = 50) there was no main effect of Group (*F*_(1,73)_ = 1.33, *p* = 0.253), Valence (*F*_(1,73)_ = 1.47, *p* = 0.229) or interaction effect (*F*_(1,73)_ = 0.008, *p* = 0.928; Figure 1). In the Acquisition phase in the CSB subjects (Reward: HV 9.39 (SD 7.34); CSB 6.39 (SD 5.43); Loss: HV 7.26 (SD 6.53); CSB 8.69 (SD 7.83)) there was a Group × Valence interaction (*F*_(1,73)_ = 4.35, *p* = 0.039) in which CSB subjects were faster to learn from Rewards and slower to learn from Losses as compared to HV. There was no Group (*F*_(1,73)_ = 0.38, *p* = 0.539) or Valence effect (*F*_(1,73)_ < 0.001, *p* = 0.983).

##### Win-stay/lose-shift

In the Lose-Shift analysis, there was a Group × Valence effect (Table 3; Figure 3); in the posthoc analysis, CSB subjects had lower Lose-Shift or were more likely to stay or perseverate after a loss in the Reward condition relative to Loss (*p* = 0.005) and Neutral (*p* < 0.001). Similarly, in the Win-Stay analysis, there was a Group × Valence effect; in the posthoc analysis, CSB had higher Win-Stay or were more likely to stay after a win in the Reward condition relative to Loss (*p* = 0.019) and Neutral (*p* = 0.007).

##### Summary

CSB subjects were faster to learning from rewards in the acquisition phase compared to HV and were more likely to perseverate or stay after either a loss or a win in the Reward condition.

#### Attentional Set Shifting

BED subjects (*n* = 31) compared to HV (*N* = 47) made more ED shift errors (*t* = −2.33, *df* = 77, *p* = 0.023). AD subjects (*n* = 30) compared to HV (*N* = 45) similarly made more ED shift errors (*t* = −3.04, *df* = 74, *p* = 0.003). In contrast, Obese subjects without BED (*N* = 30) compared to HV (*n* = 45) made a similar number of errors during the ED shift (*t* = −1.02, *df* = 74, *p* = 0.313). CSB subjects (*N* = 25) compared to HV (*N* = 37) made a similar number of errors during ED shift (*t* = 0.93, *df* = 61, *p* = 0.358). VG subjects (*N* = 24) compared to HV (*N* = 36) also made a similar number of errors during ED shifts (*t* = −0.52, *df* = 59, *p* = 0.605; Figure 2).

In AD subjects, the number of units per day was positively correlated with ED errors (reported as Pearson correlation coefficient, *p*-value: 0.394, *p* = 0.031). In Obese and BED subjects there were no correlations between ED errors and BMI or Binge Eating Severity (BES; *p* > 0.05).

## Discussion

This study provides evidence for differential cognitive flexibility impairments across different pathologies of drug and non-drug rewards as measured by two distinct behavioral paradigms: reversal learning and attentional set-shifting. Although we did not measure the neural correlates of these distinct cognitive processes in the present study, agreement on the substrates of these different tasks has evolved with the use of higher precision technology in rodent and nonhuman primate species and fMRI in humans. The literature consistently implicates differing aspects of fronto-striatal circuitry in reversal learning and attentional set-shifting, namely orbitofrontal and lateral prefrontal cortices respectively. We have previously reported on these measures of reversal learning (number of trials to criterion) and ED shifting demonstrating a relationship between dissociable fronto-striatal circuits (Morris et al., 2016).

### Reversal Learning Deficits

We found slower reversal learning in individuals with AD and VG across both reward and loss valences. Obese subjects with BED compared to those without BED also had slower reversal learning in the reward relative to loss condition whereas those without BED had slower reversal in the loss relative to reward condition.

Reversal learning impairments are common in cocaine use disorders (Camchong et al., 2011) with evidence for enhanced perseverative responding to previously rewarded stimuli (Ersche et al., 2008) but appears less prominent in studies in AD (Fortier et al., 2008; Vanes et al., 2014). Our findings converge with a study comparing AD, pathological gamblers and healthy controls which found no significant group differences in perseverative responding during reversals between alcohol-dependent patients and pathological gamblers and healthy controls but showed slower learning rates for reversal (Vanes et al., 2014). We expand on these findings demonstrating impairments in reversal trials to criterion to both reward and loss outcomes suggesting a generalized impairment in reversal learning in AD rather than a valence specific abnormality. Our version of the task is more difficult which may be more likely to induce reversal impairments. We further show that VG individuals had similar impairments in reversal learning across both valences in line with previously reported reversal learning deficits in pathological gambling, a non-substance behavioral addiction (Patterson et al., 2006).

Our findings in obese subjects with BED converge with reported preclinical and clinical findings. Rodents exposed to an unrestricted high-fat diet develop greater impairments in reversal learning (Kanoski et al., 2007). Obese mice have reduced striatal D2 receptors (Johnson and Kenny, 2010), a deficit which has been shown to impair the ability to inhibit previously rewarded responses to natural rewards in mice (Kruzich et al., 2006). In a human study of obesity, both acquisition and reversal learning were impaired specific to the food outcome but not the monetary outcome (Zhang et al., 2014). Here we focused on monetary outcomes and show that obese subjects with BED relative to those without BED were slower to learn during reversal in the reward condition but not in the loss condition. These findings suggest reversal learning impairments in BED as a function of valence. These differential effects of valence are also highly consistent with our previous report of enhanced risk taking to reward anticipation along with impaired sensitivity to reward value gradients in BED, which was not observed in obese subjects without BED (Voon et al., 2015c). Thus, these findings are similar to observations of lower goal-directed and enhanced habit formation in BED subjects relative to non-BED subjects. BED subjects may be more likely to choose actions based on expected prior rewarded actions whereas non-BED subjects may be more likely to avoid actions based on expected prior punished actions.

### Attentional Set Shifting Deficits

We further show specific impairments in ED set shifting in AD and obese subjects with BED but not in those without BED. Previous studies have shown impairments in set-shifting tested with the WCST or the Trail-making test in obese individuals with and without BED (Duchesne et al., 2010; Wu et al., 2014) and in children and adolescents with excess weight (Cserjési et al., 2007; Verdejo-García et al., 2010). In line with previous studies demonstrating impairments in set shifting in AD (Tarter, 1973; Nowakowska et al., 2007) and as a predictor of relapse (Pothiyil and Alex, 2013), we show impairments in ED set shifting correlating with alcohol severity. The findings in BED are consistent with enhanced behavioral inflexibility across multiple domains (Voon, 2015).

### Perseveration

We further measured a more basic form of behavioral inflexibility, or the tendency to stay or shift following reward or loss outcomes to assess the use of outcome valence to guide behaviors. AD subjects were also overall more likely to stay or perseverate after loss outcomes rather than shift across both acquisition and reversal arms compared to HV suggesting an impaired ability to integrate loss outcomes to guide behaviors. In the comparison with Obese subjects without BED, those with BED were more likely to stay following larger rewarding outcomes consistent with impaired reversal learning specific to the reward condition.

### Study Limitations

One limitation of the present study is the lack of direct confirmation of diagnostic in all the disorders assessed. We used alcohol breathalyzer tests on the day of testing to make sure the participants were not alcohol free to confirm abstinence in AD participants. However, a similar measure was not used in other groups where use or compulsive behavior cannot be easily confirmed in such a direct way, which may be seen as the alcohol dependent group being treated differently than the other groups. However, an extensive interview assessment was employed and all diagnosis was made by an experienced psychiatrist, which makes us believe that limitation was addressed. A second limitation is the fact that, in the reversal learning task, participants were instructed that they would be choosing from three different pairs of symbols but at some point the relationship between the symbols and the likelihood of winning and not losing money might change. This instruction may alter the way individuals solve the task and could change brain recruitment, redirecting from a model-free to a model-based learning strategy. However, if a model-based strategy was more predominant as consequence of the instruction, the likelihood of finding impairments in this task would be lower. Since the direction of the effect of a model-based strategy is opposite of our results, we do not think this limitation affected our findings in any way.

### Behavioral Flexibility Deficits Across Disorders of Compulsivity

In Table 4 we summarize these results and studies on compulsivity indices within this same human patient cohort. Compulsivity has multiple subtypes with overlapping yet discrete neural substrates (Voon and Dalley, 2015). Here we define compulsivity and behavioral flexibility on the basis of flexible responding to context and can be conceptualized hierarchically from more complex to basic. Our previous studies have also described shifts in reinforcement learning in the context of task structure or goals (model-based goal-directed learning) relative to previously reinforced choices (model-free habitual learning; Voon et al., 2015b) and explore-exploit behaviors in the context of uncertainty (Morris et al., 2015). These compulsivity subtypes implicate lateral prefrontal and medial orbitofrontal and anterior prefrontal cortices respectively. In this current study, contextual changes may occur to a higher level explicit rule shift in attentional set shifting or to changes in reinforcement contingencies in reversal learning (Chase et al., 2011) implicating lateral prefrontal and orbitofrontal cortices respectively. More basic forms of behavioral inflexibility include perseverative responding either following valenced feedback or irrespective of feedback (Voon et al., 2015b).

**Table 4:** **Summary of compulsivity measures across disorders**.

	Brain regions (Morris et al., 2015; Voon et al., 2015b)	AD	Obese + BED	Obese − BED	VG	CSB
Exploration-exploitation (Morris et al., 2015)	Anterior PFC	+	+ (Reward)	+ (Loss)	–*	?
Attentional set shifting	LPFC	+	+	–	–**	–
Goal-directed/ habit (Voon et al., 2015b)	DLPFC Medial OFC	+ (early abstinence)	+	–	–*	?
Reversal	OFC	+	+ (Reward)	+ (Loss)	+	–
Perseveration	?	+ (Loss)	+ (feedback) independent; Voon et al. (2015b) + (Reward)	–	–	+ (Reward)

+ Represents enhanced compulsivity (i.e., decreased exploration/greater exploitation; impaired set shifting; decreased goal-directed/greater habit; impaired reversal; greater perseveration); – Represent no difference from comparator group. *Data not shown. **Reports data from one of our studies not yet published. We are not considering here other set shift studies. Abbreviations: AD, alcohol dependence; BED, binge eating disorder; VG, pathological video gaming; CSB, compulsive sexual behavior; PFC, prefrontal cortex; DL, dorsolateral; OFC, orbitofrontal cortex.

Together these findings suggest marked similarities in impairments across multiple forms of behavioral flexibility in AD and BED (Voon, 2015) across both orbitofrontal and lateral prefrontal cortices. Obese subjects with BED exhibit a wider range of impairments of behavioral flexibility relative to those without BED with the former demonstrating greater sensitivity to reward valence and the latter to loss valence. Deficits observed in VG focus on the orbitofrontal cortex (OFC), suggesting a commonality across drug and non-drug addictions.

In contrast to other disorders, CSB compared to HV showed faster acquisition to reward outcomes along with a greater perseveration in the reward condition irrespective of outcome. The CSB subjects did not show any specific impairments in set shifting or reversal learning. These findings converge with our previous findings of enhanced preference for stimuli conditioned to either sexual or monetary outcomes, overall suggesting enhanced sensitivity to rewards (Banca et al., 2016). Further studies using salient rewards are indicated. Deficits in goal-directed or exploratory behaviors in CSB have not yet been reported.

Our findings have several important implications. First, these observations may be interpreted as commonalities across AD and BED supporting pathological binge eating behaviors in BED as a possible form of behavioral addiction. Alternatively, the degree of behavioral inflexibility may also be representative of severity. Second, commonalities across OFC reversal deficits may link these pathological drug and non-drug behaviors. Third, we emphasize the differential influence of valence on behavioral flexibility, particularly emphasizing its relevance in obesity with and without bingeing and CSB. Finally, our findings highlight compulsivity and behavioral inflexibility as a relevant domain in the trend towards defining relevant cognitive endophenotypes dimensionally across psychiatric disorders (Insel et al., 2010).

## Author Contributions

VV was responsible for the study concept and design. PB and VV contributed to the acquisition of the data, performed the data analysis and wrote the article. All authors critically reviewed content and approved final version for publication.

## Conflict of Interest Statement

The authors declare that the research was conducted in the absence of any commercial or financial relationships that could be construed as a potential conflict of interest.
